# RPE Adenocarcinoma as the Presenting Sign of Bronchogenic Carcinoma: Diagnostic Dilemma in the Management of a Case

**DOI:** 10.1155/2013/786378

**Published:** 2013-03-27

**Authors:** Namrata Adulkar, Santhi Radhakrishnan, N. Vidhya, Usha Kim

**Affiliations:** ^1^Department of Orbit, Oculoplasty & Ocular Oncology, Aravind Eye Hospital & Postgraduate Institute, Madurai, Tamil Nadu 652020, India; ^2^Department of Ocular Pathology, Aravind Eye Hospital & Postgraduate Institute, Madurai,Tamil Nadu 652020, India

## Abstract

Neoplasms of retinal pigment epithelium are rare and must be differentiated from choroidal melanoma. The possibility of a metastatic disease with possible primary sites as lung, breast, or kidney should be ruled out. Herein we report a case of adenocarcinoma arising from the RPE with a lung lesion suspicious of bronchogenic carcinoma. In this paper, ocular symptoms were the first sign of a systemic malignancy.

## 1. Introduction 

Patients with systemic malignancy present to an ophthalmologist primarily with ocular complaints in 12–31% cases. Herein we report a case of adenocarcinoma arising from the retinal pigment epithelium with a lung lesion suspicious of bronchogenic carcinoma.

## 2. Case Report 

**Figure 1:**
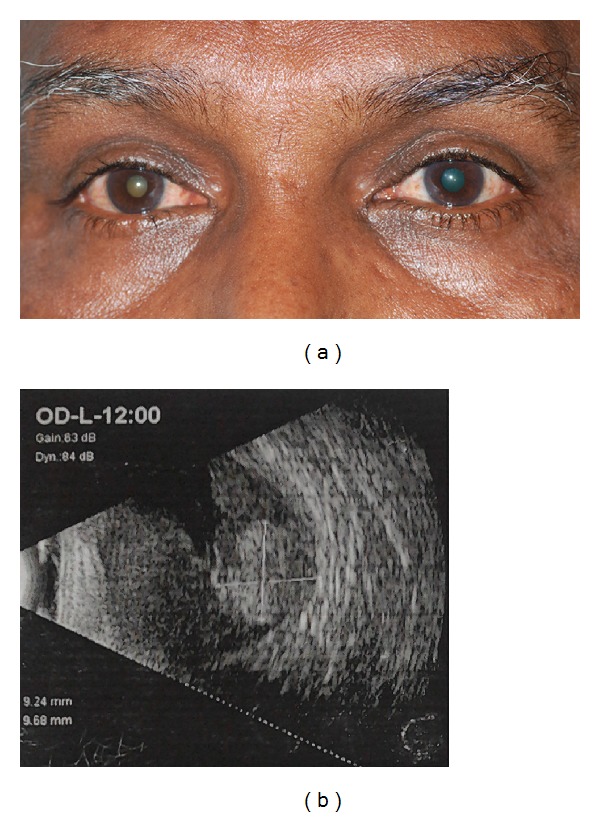
(a) External color photograph of the patient showing white reflex in the right eye. (b) Ultrasound B-scan showing mushroom-shaped mass superonasal to the optic disc with total exudative retinal detachment.

A 65-year-old gentleman, a retired cotton mill worker, presented to our clinic with right eye diminution of vision of 10 days duration. He was also a smoker for the past 45 years but was otherwise fit. On examination, his best corrected visual acuity was no perception of light in OD and OS was 6/9, N6. He had an afferent pupillary defect in the right eye, and his fundus examination revealed a total exudative retinal detachment with a mass lesion in the superonasal quadrant of posterior retina. B-scan ultrasonography showed a 9.68∗9.24 mm mushroom-shaped mass lesion with high internal reflectivity and acoustic solidity, superonasal to the optic disc with total exudative retinal detachment (Figure 1). Computed tomography scan of the orbit revealed an intraocular mass lesion over optic disc in the superonasal quadrant. There was no evidence of optic nerve infiltration or breach of scleral coats. A working diagnosis of choroidal melanoma was made. Of the treatment options offered, the patient opted for enucleation of the eye. 

**Figure 2:**
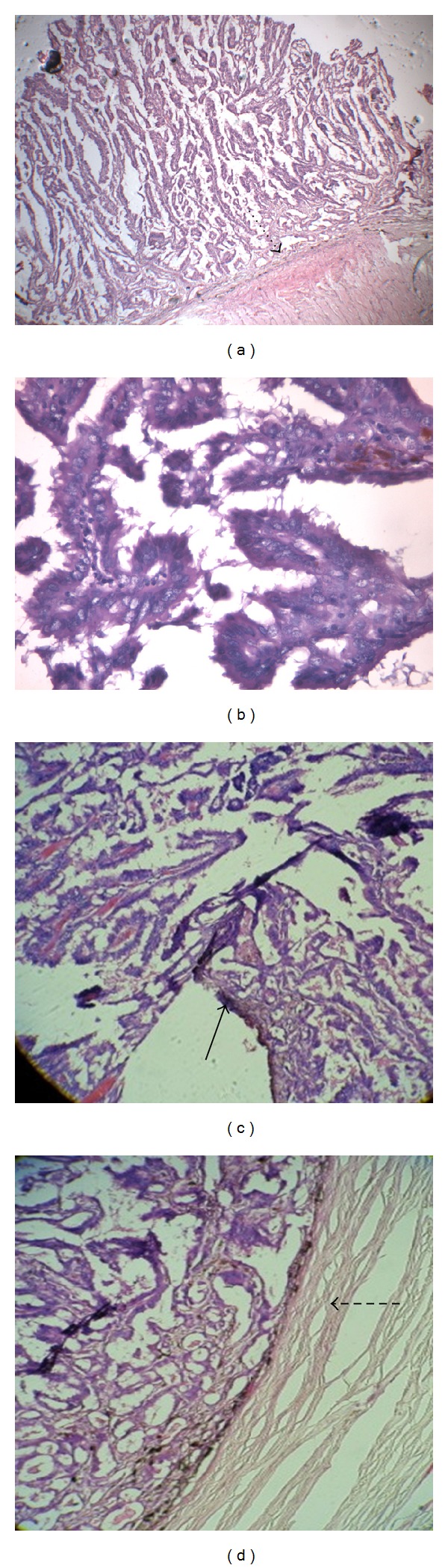
Histopathological photomicrograph (H & E stain) showing (a) low magnification (4x) intraocular tumor at posterior pole of the globe arising from the RPE sitting over the optic nerve head (dotted arrow) and infiltrating into the choroid. (a) High magnification (40x) typical papillary pattern with round to oval cells with vesicular nuclei suggestive of adenocarcinoma. Focal areas showing pigmentation are seen. (c) Tumor seen breaking through the RPE (arrow) and infiltrating the choroid. (d) Tumor arising from the RPE and involving the optic nerve (dashed arrow).

**Figure 3:**
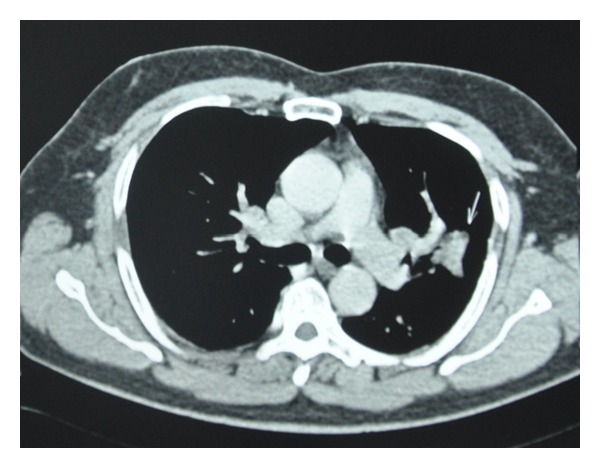
CT scan of the lung (transverse section) showing well-defined nodular lesion measuring 4.1∗3.6 cms in the left upper lobe with spiky margins.

**Figure 4:**
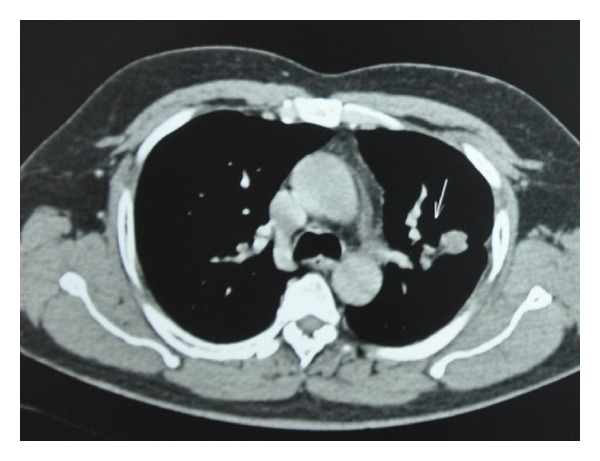
After 4 cycles of chemotherapy CT scan of the lung (transverse section) showing reduction in the size of the lung lesion; measuring 3.0∗2.1 cms in the left upper lobe.

The histopathological examination of the enucleated eyeball revealed a well-circumscribed tumor arising from the RPE and composed of round-to-oval cells with vesicular nuclei arranged in papillary pattern with fibrovascular core and mucinous stroma (Figure 2). Numerous mitoses were seen. The tumor cells showed positive immunostaining for cytokeratin and vimentin suggesting epithelial origin of the tumor and negative for melanin-specific HMB-45. This was suggestive of a well-differentiated adenocarcinoma of the retinal pigment epithelium and infiltrating the vitreous, choroid, and optic nerve head. Given the rarity of this tumor, a high suspicion index for a secondary origin was maintained. Metastatic workup identified a mass lesion in the lung on high-resolution computed tomography scan which pointed towards the diagnosis of primary bronchogenic carcinoma (Figure 3). The patient was referred to an oncologist and was advised a bronchoscopic biopsy of the lung lesion. However, the patient refused any intervention at this point in time. He was treated with chemotherapy with mitomycin C, vinblastine, and prednisolone. After 4 cycles of chemotherapy given at 3 weeks interval, the size of the lung lesion reduced considerably indicating favorable response to therapy (Figure 4).

## 3. Comment 

Primary ocular adenocarcinoma arising from neuroepithelium is extremely rare, and the possibility of a secondary adenocarcinoma with possible primary sites as lung, breast, or kidney should be ruled out [1]. Essentially all reported examples of adenocarcinomas of the RPE have been enucleated with a presumptive diagnosis of uveal melanoma [2].

Shields et al. [3] describe retinal pigment epithelial tumors as dark-coloured tumors of variable size, with no particular predilection for posterior pole or retinal periphery, and that its differentiation from a choroidal melanoma can be challenging. Clinical presentations include visual loss due to exudative retinal detachment or vitreous hemorrhage. These malignancies tend to exhibit local invasiveness into the choroid and sensory retina, and rarely a RPE adenocarcinoma with extrascleral extension may have distant metastases [4]. 

Ocular metastases were historically considered as a rare finding; however, it is now recognized that choroidal secondaries are the most common intraocular neoplasia. Choroid has been reported to be the most common site of intraocular metastases [5]. Lung is the commonest site of primary disease in male patients with ocular metastases [6], most common histologic type is adenocarcinoma followed by small cell and squamous cell carcinoma [5]. However, the initial sign of lung cancer may rarely be visual symptoms due to ocular metastasis. In such a condition, the lung cancer is already at, an advanced stage, and the prognosis is poor [5]. However, our patient was absolutely asymptomatic for the lung disease and imaging showed a lung lesion suggestive of early disease. This raised the dilemma as to whether it is the primary lesion or a secondary, which remains undetermined due to the lack of histological diagnosis of lung lesion. Although the history of smoking for 40 years and cotton mill worker may suggest an increased likelihood of a primary lung lesion, the causative association cannot be assumed.

Bronchogenic carcinoma is known to respond to chemotherapy and is the standard treatment of choice. Our patient too showed good response to chemotherapy with reduction in size of the lung lesion after 4 cycles of chemotherapy.

To conclude, this is a rare case of intraocular adenocarcinoma with an associated bronchogenic lesion. In such patients, a systematic search for the primary lesion is required and histological diagnosis of the extraocular lesion is mandatory to confirm the nature of the disease.
